# TRANSLATION, RELIABILITY, AND VALIDITY OF THE TRUNK IMPAIRMENT SCALE IN A POLISH POPULATION AFTER STROKE

**DOI:** 10.2340/jrm.v58.44789

**Published:** 2026-03-10

**Authors:** Joanna MAŁECKA, Magdalena GOLIWĄS, Katarzyna ADAMCZEWSKA, Jacek LEWANDOWSKI, Geert VERHEYDEN, Dawid ŁOCHYŃSKI

**Affiliations:** 1 Department of Neuromuscular Physiotherapy, Poznan University of Physical Education, Poznan; 2 Department of Musculoskeletal Rehabilitation, Poznan University of Physical Education, Poznan, Poland; 3 Department of Rehabilitation Sciences, KU Leuven – University of Leuven, Leuven; 4 Leuven Brain Institute, KU Leuven – University of Leuven, Leuven, Belgium

**Keywords:** stroke, outcome measure, TIS, FMA, ARAT, trunk impairment

## Abstract

**Objective:**

This study was conducted to estimate the reliability and validity of the translated and culturally adapted Polish version of the Trunk Impairment Scale in post-stroke patients and to determine the strength of the relationship between trunk and upper limb physical function after stroke.

**Design:**

The Polish version of the Trunk Impairment Scale was developed through cultural adaptation. Internal consistency, test–retest reliability, and inter-rater reliability were determined, and construct validity was evaluated by analysing the Polish version of the Trunk Impairment Scale, the Polish version of the Fugl-Meyer Assessment Scale, and the Polish version of the Action Research Arm Test.

**Participants:**

Eighty patients with diagnosed stroke in the subacute and chronic stages.

**Results:**

The internal consistency for the Polish version of the Trunk Impairment Scale was excellent (α = 0.85–0.91). Test–retest and inter-rater reliability were almost perfect (ICC = 0.94–1.0, κ = 0.92–1.0). Construct validity was moderate (rho = 0.71–0.76). A moderate correlation was also found between the Polish version of the Trunk Impairment Scale and Polish version of the Action Research Arm Test total scores (rho = 0.60).

**Conclusion:**

The Polish version of the Trunk Impairment Scale is a reliable and moderately valid outcome measure to assess trunk impairment in Polish stroke survivors. Trunk function is moderately related to gross and fine motor skills of the arm, hand, and fingers among individuals with stroke.

## LAY ABSTRACT

Two primary objectives were pursued in this study. The first aim was to evaluate the diagnostic utility of the newly developed Polish version of the Trunk Impairment Scale. The second was to examine the relationship between trunk and upper extremity function. A total of 80 post-stroke participants were enrolled. The Trunk Impairment Scale was translated into Polish, and subsequently, the reliability and validity of the Polish version of the Trunk Impairment Scale were calculated. Furthermore, the correlation between the Polish version of the Trunk Impairment Scale and the Polish version of the Action Research Arm Test was analysed. The results showed that the Polish version of the Trunk Impairment Scale is a highly reliable and moderately valid outcome measure for assessing individuals in the Polish population after stroke. Moreover, a moderate relationship between trunk function and upper extremity activity was demonstrated.

Trunk control is the main indicator of functional recovery after stroke (1, 2).There is a link between trunk performance, balance, gait, and functional outcome in stroke patients (3, 4). Physical rehabilitation of trunk function is essential; thus, reliable and valid outcome measures are critical for assessing the level of trunk function impairment in clinical practice.

The trunk plays an integral role in postural stability by supporting controlled movement of the extremities during motor tasks (5, 6). The enhancement of trunk stability and control is regarded as a fundamental requirement for upper extremity function and effective hand use (7). Trunk stability affects shoulder stability, thereby enhancing elbow, wrist, and finger motion. A stable trunk establishes a firm base for the force produced by the extremities (8). It has previously been reported that trunk physical function is linked to upper extremity functional performance after stroke (4, 9, 10, 11). Thus, it is important to assess upper extremity function in addition to trunk performance.

The Trunk Impairment Scale (TIS) was designed to assess motor impairment of the trunk after stroke (12). The TIS has been translated into several languages in previous studies (1, 13, 15–20), and this outcome measure has demonstrated good psychometric properties (1, 14–19). The TIS has demonstrated low to excellent internal consistency in patients after stroke (α = 0.61–0.97) (1, 14, 15, 18, 20). The test–retest reliability, calculated using intraclass correlation coefficient values, is moderate to excellent (*r* = 0.73–0.97) (1, 5, 6, 9). The inter-rater reliability values for the total and subscale scores range from 0.63 to 0.99 in post-stroke patients (1, 14, 15, 18). Studies examining the validity have demonstrated moderate, good, or high (19, 20) correlations (rho = 0.56–0.86) between the TIS and the total scores of other outcome measures, such as the Trunk Control Test, Barthel Index, Berg Balance Scale, Fugl Meyer Assessment Balance, and Rivermead Mobility Index (12, 18, 19).

There is no culturally adapted and validated Polish tool specifically designed to assess trunk physical function that could establish the construct validity of the TIS. Furthermore, the relationship between the control of selective trunk movements and the fine control (e.g., pinching, grasping) of the upper extremities has never been studied using dedicated measurement tools such as the TIS and ARAT (21). Both outcome measures are considered adequate for assessing postural control of the trunk (12) and dexterity of upper-extremity movements after stroke (22). Thus, assessing these factors may provide a new perspective on therapeutic strategies to improve trunk control and upper limb function after stroke. The present study was conducted to estimate the reliability and validity of the translated and culturally adapted Polish version of the TIS in a population of post-stroke patients. The construct validity of this newly adapted tool was determined by verifying the strength of correlation between the TIS-PL and the Polish version of the Fugl-Meyer Assessment Scale (FMA-PL) total and subscale scores (23). The second aim was to determine the correlation between the TIS-PL and ARAT-PL to verify the strength of the association between trunk function and upper extremity activity.

## METHODS

### Study design, participants, initial evaluation

In this 7-month cross-sectional study, 80 patients with stroke were recruited from the Bonifraterskie Centrum Zdrowia hospital in Piaski, Poland. The inclusion criteria were as follows: (*i*) a diagnosis of stroke, indicated by computed tomography scans or magnetic resonance imaging, (*ii*) the presence of hemiparesis, and (*iii*) no additional neurological or orthopaedic disabling deficits. The exclusion criteria were (*i*) hemiplegia in the upper extremity (assessed by a neurologist), (*ii*) serious visual and hearing disorders, (*iii*) cognitive decline that limited administration of the tests, (*iv*) disorders of speech and language, (*v*) native language other than Polish, and (*vi*) hip prosthesis.

The data collected in the initial evaluation included age, sex, weight, height, and upper limb dominance. Furthermore, data on time post-stroke, lesion type, lesion location, involved side of the lesion, presence of comorbidities, and duration of hospital rehabilitation were collected. Ethical approval (187/19) was obtained from the Bioethical Committee of Poznan University of Medical Sciences, and the study was conducted in accordance with the tenets of the Declaration of Helsinki. All participants provided informed consent at the time of enrolment in the study.

### Outcome measures

*TIS.* The TIS comprises 3 subscales: Static sitting balance, Dynamic sitting balance, and Coordination. The 3 subscales comprise 3, 10, and 4 items, respectively. The scale scores range from 0 to 23, with higher scores indicating better trunk control. The total scores of the Static sitting balance, Dynamic sitting balance, and Coordination sections are 7, 10, and 6, respectively. Participants are required to maintain the sitting position for at least 10 s for testing (12).

*FMA.* The FMA was investigated to evaluate sensorimotor impairments in patients with stroke (24). This measure comprises 5 domains: motor functioning, balance, sensation, joint range of motion, and joint pain (24). The Polish version of FMA was used in this study (23). Only the motor domain of the Polish version of the FMA for the upper (FMA-UE-PL) and lower (FMA-LE-PL) extremities was administered (23). The motor score is expressed on a 3-point ordinal scale: 2 points indicate the task was fully performed, 1 point indicates it was partially performed, and 0 points indicate it was not performed. The maximum scores for the FM-UE and FM-LE are 66 and 34 points, respectively (25–27).

*ARAT.* The ARAT assesses upper-limb activity capacity according to the International Classification of Functioning, Disability and Health (ICF), specifically dexterity and object-handling abilities. The ARAT consists of 19 items across the subtests Grasp, Grip, Pinch, and Gross Movement. The subtest Grasp and Pinch comprises 6 items, each. The subtests Gross Movement and Grip comprise 3 and 4 items, respectively. The total ARAT score is the sum of the scores of 19 items. The maximum attainable score is 57 points. Each functional task is assigned an ordinal score of 0, 1, 2, or 3 points. Higher values indicate better upper limb functional status (22).

### The TIS translation and cultural adaptation

The TIS was translated into Polish in accordance with international guidelines (28–31). First, we obtained permission to proceed with cultural adaptation and validation from the author of the original TIS (12). The English version of the TIS was then independently translated by 2 Polish translators fluent in English (1 specialized in the field of rehabilitation). Two Polish versions were collated; differences between the translations were discussed, corrected, and a common draft version was jointly established. In the next stage, this Polish draft was independently back-translated into English by 2 certified English translators. The common retranslated English version was then compared with the original English version by 2 physiotherapists fluent in English. Corrections were made to the retranslated version, and a panel of judges consisting of a neurologist, 2 physiotherapists specialized in neurology, 1 in clinical neurophysiology, and 1 in orthopaedics, a psychologist, and translators compared and discussed the translated and original versions of the TIS. The emerging Polish version of TIS was corrected to account for all differences, achieving a satisfactory balance between cultural language requirements and the original English scale, yielding the pre-final version of the TIS-PL. This version was then applied in a pilot study with a small group of participants and declared final.

### Assessment procedure

The 2 experienced neurological physiotherapists, well trained in administering the scales, carried out the TIS-PL, FMA-UE-PL, FMA-LE-PL, and ARAT-PL. To determine test–retest reliability, the same rater examined the patients twice a day, with a 2-h gap between assessments. Inter-rater reliability was assessed by 2 observers who independently examined patients simultaneously in a silent hospital room (32). Results were collected for the total and subscale scores of the TIS-PL, FMA-PL, and ARAT-PL.

### Statistical analysis

The statistical analysis was conducted using the statistical software package Statistica 13 (Tibco Software Inc, 2017, Polska, Kraków, Poland) (33) and RStudio (psych package version 2.4.3; R Foundation for Statistical Computing, Vienna, Austria) (34).

*Internal consistency.* Internal consistency was evaluated by calculating Cronbach’s alpha coefficients for the subscales and the total scale. The Cronbach’s alpha values indicate excellent, adequate, and poor results as above 0.80, 0.70–0.79, and below 0.70, respectively (35, 36).

*Reliability.* The test–retest and inter-rater reliability of the TIS-PL were estimated using Cohen’s kappa (κ), the intraclass correlation coefficient (ICC), and percentage agreement (PA). Item reliability was determined when agreement exceeded 80% (37). Cohen’s kappa values can range from 0 (no agreement) to 0.01–0.20 (none to slight), 0.21–0.40 (fair), 0.41–0.60 (moderate), 0.61–0.80 (substantial), and 0.81–1.00 (almost perfect agreement) (38). The correlation coefficients range from 0–0.30, indicating little or no correlation between the studied variables; from 0.30–0.50, indicating low correlation; from 0.50–0.70, indicating moderate correlation; from 0.70–0.90, indicating high correlation; and from 0.90–1.00, indicating very high correlation (20).

*Validity.* Construct validity was assessed using hypothesis testing according to the guidelines of the COnsensus-based Standards for the selection of health Measurement INstruments (COSMIN) (30). A total of 8 independent hypotheses were formed. For all the hypotheses, we defined the correlation direction, the correlation strength (calculated as the Spearman rank correlation coefficient, rho), and the rationale for each (Tables I and II). The relationships among TIS-PL, FMA-UE-PL, and ARAT-PL scores (4 hypotheses for each) and FMA-LE-PL scores (4 hypotheses) were examined to estimate the extent to which the observed correlations are consistent with the formulated hypotheses. The TIS-PL construct validity rating was determined according to the total number of confirmed hypotheses; 6–8 (≥ 75%) confirmed hypotheses indicated high construct validity, while 4–5 (50% ≥) indicated moderate (30).

**Table I:** Method of hypothesis testing used to assess the construct validity of the TIS-PL according to its associations with the FMA-UE-PL and ARAT-PL

Hypotheses tested	Rationale	Correlation expected	FMA-UE-PL		ARAT-PL	
			Correlation actual	Confirmed?	Correlation actual	Confirmed?
1. There will be at least a moderate–strong positive correlation between the overall result of the TIS-PL, FMA-UE-PL, and ARAT-PL	The TIS-PL, FMA-UE-PL, and ARAT-PL measure similar constructs (the relationship between actual postural control and upper limb motor performance)	≥ 0.50	0.71	Yes	0.61	Yes
2. There will be at least a moderate–strong positive correlation between the result of the TIS-PL subtest Static sitting balance and the total FMA-UE-PL and ARAT-PL result	The TIS-PL, FMA-UE-PL, and ARAT-PL measure similar constructs (the relationship between actual static sitting balance and upper limb motor performance)	≥ 0.50	0.42	No	0.31	No
3. There will be at least a moderate–strong positive correlation between the result of the TIS-PL dynamic sitting balance subtest and the total FMA-UE-PL and ARAT-PL scores	The TIS-PL, FMA-UE-PL, and ARAT-PL measure similar constructs (the relationship between actual dynamic sitting balance and upper limb motor performance)	≥ 0.50	0.72	Yes	0.68	Yes
4. There will be at least a moderate–strong positive correlation between the result of the TIS-PL coordination subtest and the total FMA-UE-PL and ARAT-PL scores.	TIS-PL and FMA-UE-PL measure similar constructs (the relationship between actual coordination and upper limb motor performance)	≥ 0.50	0.62	Yes	0.50	Yes

TIS-PL: Polish version of the Trunk Impairment Scale; FMA-UE-PL: Polish version of the Fugl-Meyer Assessment for Upper Extremity; ARAT-PL: Polish version of the Action Research Arm Test.

**Table II:** Method of hypothesis testing used to assess the construct validity of the TIS-PL according to its associations with the FMA-LE

Hypotheses tested	Rationale	Correlation expected	FMA-LE-PL	
			Correlation actual	Confirmed?
6. There will be at least a moderate–strong positive correlation between the overall result of the TIS-PL and FMA-LE-PL	The TIS-PL and FMA-LE-PL measure related constructs (the relationship between postural control and lower limb motor performance)	≥ 0.50	0.76	Yes
7. There is at least a moderate–strong positive correlation between the result of the TIS-PL subscale Static sitting balance and the total result of the FMA-LE-PL	The TIS-PL and FMA-LE-PL measure related constructs (the relationship between postural control and lower limb motor performance)	≥ 0.50	0.39	No
8. There is at least a moderate–strong positive correlation between the result of the TIS-PL subscale Dynamic sitting balance and the total FMA-LE-PL result	The TIS-PL and FMA-LE-PL measure related constructs (the relationship between postural control and lower limb motor performance)	≥ 0.50	0.69	Yes
9. There is at least a moderate–strong positive correlation between the result of the TIS-PL co-ordination subscale and the FMA-LE-PL total score.	The TIS-PL and FMA-LE-PL measure related constructs (the relationship between postural control and lower limb motor performance)	≥ 0.50	0.69	Yes

TIS-PL: Polish version of the Trunk Impairment Scale; FMA-LE – PL: Polish version of the Fugl-Meyer Assessment for Lower Extremity.

*Relationship between trunk and upper extremity.* According to the literature (4, 10, 11, 39–42), there is a link between trunk and upper extremity function. Thus, we hypothesized that a significant correlation between the TIS and the ARAT would be present. This correlation was evaluated by calculating Spearman’s rank correlation coefficients for the relationship between the ARAT-PL (43) and the TIS-PL. Correlation is considered low when the calculated coefficient is between 0.30 and 0.50, moderate when it is between 0.50 and 0.70, high when it is between 0.70 and 0.90, and very high when it is between 0.90 and −1.00 (44).

*Floor and ceiling effects.* Floor and ceiling effects were established as the percentages of participants who scored beyond the lower (floor) and upper (ceiling) boundaries of the total TIS-PL score (0–23).

The cut-off points for these boundaries were set at 5%. Therefore, scores under 1 were determined as the floor, while those above 22 were considered ceiling effects. Floor and ceiling effects were considered significant if more than 20% of the participants fell outside the set lower or upper boundaries, respectively (45).

## RESULTS

### Patients’ clinical characteristics

Eighty patients with subacute and chronic stages of stroke participated in the examination. The patient demographics are presented in Table III.

**Table III:** Demographics (*n* = 80)

Variable	Result
Age, mean ± SD (range)	63 ± 12.3 (31–85)
Days since stroke, mean ± SD (range)	46 ± 43.4 (10–313)
Female/male, %	37.5%/62.5%
Left/right hemiplegia, %	58.8%/41.2%
Upper limb dominance: right-/left-/both-handed, %	95%/0%/5%

SD: standard deviation, range (min-max).

### Translation and cultural adaptation

*Forward, backward, and final versions of the translation.* Several linguistic differences were detected during the first review of the Polish version of TIS translated from English. These differences mainly concerned contextual variations in the clarification or manner of expressing concepts. With few exceptions, Polish is characterized by a more long-winded style of expression and is richer in words with variable, very specific meanings. Therefore, the concise or slender English phrases or sentences were supplemented or replaced with more detailed and elaborated Polish ones. Some discrepancies were found when the original version was compared with the common version resulting from 2 back translations. As expected, all words added in the Polish version during forward translation were also added in the back translation. During the panel meeting, all versions of the survey were compared; whenever any language doubt arose, specific stylistic corrections were made, and sentences were rephrased to make the text more comprehensible to general respondents (Table IV).

**Table IV:** Changes introduced during the process of cultural adaptation

Forward translation		Back translation		Final translation	
Original words/sentences	Translated words/sentences	Original words/sentences	Translated words/sentences	Original words/sentences	Translated words/sentences
Trunk Impairment Scale	Trunk Dysfunction Scale	The arms rest on the legs	The arm was placed on the lap	Trunk Dysfunction Scale	Trunk Disability Scale
Item	Task	Are	Are set	If needed	If it is needed
Arm	Upper limb	Static sitting balance	Static stability while seated	Intermediate	Midline
Make full contact	Adhere fully	Practice	Training	Upper limb	Upper limbs
Placed	Positioned	Upper limb	Arm	Unaffected	Indirectly affected
Knee	In the knee joint	Displaces	Leans		
The arms rest on the legs	Upper limbs are placed on thigh	Knee flexion	Flexion in the knee joint		
If hypertonia is present the position of the hemiplegic arm is taken as the starting position	If hypertension occurs the position of the hemiplegic upper limb is recognized as starting position	Dynamic sitting balance	Dynamic balance while seated		
Midline	Intermediate				
Are	Are positioned				
Scores	Obtain				
Is	Amount to				
No	Has been deleted				
Static sitting balance	Static stability in sitting				
Starting position	Initial position				
Crosses	Put on				
Displaces	Swing away				
Assist crossing with the hand	When putting on the lower limb help yourself with the hand				
Dynamic sitting balance	Dynamic stability in sitting				
Is instructed	Receive a command				
Limb	Hemiplegic limb				
Patient demonstrates no or opposite shortening/lengthening	There is no body shortening/lengthening on the patient, or it occurs in the reverse way				
Patient demonstrate appropriate shortening/lengthening	There is appropriate body shortening/lengthening				
Ipsilateral	At the same side				
Upper trunk	Upper part of the trunk				
Is not moved	Is not moved forward				
Lower trunk	Lower part of the trunk				
Fixated	Remain				

### Reliability

The mean scores of the TIS-PL, FMA-UE-PL, and FMA-LE-PL obtained from the participants are presented in Table V. Differences in scores between raters for each TIS-PL assessment are presented in Table VI.

**Table V:** Total performance scores obtained from the first assessment by rater 1 for the studied outcome measures (*n* = 80)

Variable	Score (points)	Q1	Q3
TIS-PL	20 ± 4.1 (0–23)	48	65.5
FMA-UE-PL	53 ± 20.2 (0–66)	23.5	33
FMA-LE-PL	27 ± 8.2 (0–34)	17	23
ARAT-PL	8 ± 2.9 (0–57)	27.5	57

The values are presented as mean ± SD (min–max), Q1: lower quartile; Q3: upper quartile; TIS-PL: Polish version of the Trunk Impairment Scale; FMA-UE-PL: Polish version of the Fugl-Meyer Assessment for Upper Extremity; FMA-LE: Polish version of the Fugl-Meyer Assessment for Lower Extremity.

**Table VI:** Difference in scores obtained from raters for each TIS assessment (*n* = 80)

Variable	Difference in scores (rater 1, 2 tests)	Difference in scores (rater 1 and rater 2)
Static sitting balance	0.04 ± 0.34	0.04 ± 0.46
Dynamic sitting balance	0.01 ± 0.11	−0.03 ± 0.42
Co-ordination	0.00 ± 0.00	−0.04 ± 0.30
Total TIS	0.04 ± 0.34	−0.01 ± 0.54

The values are presented as mean ± SD.

*Internal consistency.* The TIS-PL total score demonstrated excellent internal consistency (Cronbach’s alpha = 0.89). Equivalently, Cronbach’s α values for the subscale scores were excellent (α = 0.85–0.91).

*Test–retest.* The test–retest reliability, as measured by kappa values, for the TIS-PL items’ scores was almost perfect (Table VII). The ICC (2,k) values determined for each subscale and the total score ranged from 0.94 to 1.00, showing excellent reliability (Table VIII).

**Table VII:** Kappa, weighted kappa, and percentage agreement values for the test–retest and inter-rater reliability (*n* = 80)

Item	Test–retest			Inter-rater		
	Қ / қ w	Value	PA	Қ / қ w	Value	PA
Static sitting balance						
Item 1	қ	1.00	100%	қ	1.00	100%
Item 2	қ	0.98	98.75%	қ	0.97	98.75%
Item 3	қ w	0.97	98.75 %	қ w	0.92	97.50%
Dynamic sitting balance						
Item 4	қ	1.00	100%	қ	0.93	98.75%
Item 5	қ	1.00	100%	қ	1.00	100%
Item 6	қ	1.00	100%	қ	0.93	96.25%
Item 7	қ	1.00	100%	қ	0.66	98.75%
Item 8	қ	1.00	100%	қ	0.93	97.50%
Item 9	қ	1.00	100%	қ	0.84	97.50%
Item 10	қ	1.00	100%	қ	0.94	97.50%
Coordination						
Item 1	қ w	0.98	98.75%	қ w	1.00	100%
Item 2	қ	1.00	100%	қ	0.95	97.50%
Item 3	қ w	1.00	100%	қ w	0.96	97.50%
Item 4	қ	1.00	100%	қ	1.00	100%

Қ: kappa value; қ w: weighted kappa value; PA: percentage agreement.

**Table VIII:** Test–retest reliability (*n* = 80)

Subtest scores (points)	Lower CI (95%)	ICC	Upper CI (95%)
Static sitting balance (0–7)	0.908	0.936	0.936
Dynamic sitting balance (0–10)	0.999	0.999	0.999
Co-ordination (0–6)	1.000	1.000	1.000
Total TIS-PL (0–23)	0.996	0.998	0.998

CI: confidence interval; ICC: intraclass correlation coefficient; TIS-PL: Polish version of the Trunk Impairment Scale.

*Inter-rater*. The TIS-PL subscale items exhibited moderate to almost perfect κ values, ranging from 0.66 to 1.0 (Table VII). The ICC (2,k) values for each subscale and the total instrument score ranged from 0.81 to 0.99, indicating good to excellent reliability (Table IX).

**Table IX:** Inter-rater reliability (*n* = 80)

Subtest scores (points)	Lower CI (95%)	ICC	Upper CI (95%)
Static sitting balance (0–7)	0.807	0.867	0.867
Dynamic sitting balance (0–10)	0.985	0.989	0.989
Co-ordination (0–6)	0.992	0.995	0.995
Total TIS-PL (0–23)	0.994	0.996	0.996

CI: confidence interval; ICC: intraclass correlation coefficient; TIST-PL: Polish version of the Trunk Impairment Scale.

### Validity

**Fig. 1:**
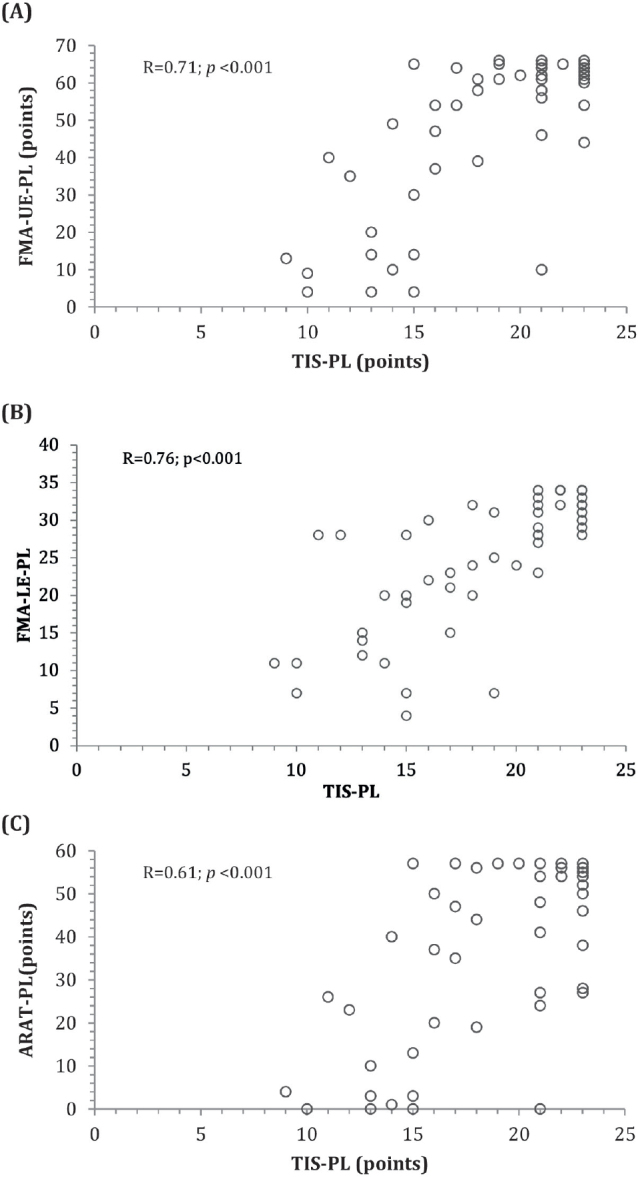
Relationship between (A) the TIS-PL and FMA-UE scores, (B) the TIS-PL and FMA-LE scores, and (C) the TIS-PL and ARAT-PL scores.

The relationships between the scores of the TIS-PL, FMA-UE-PL, and FMA-LE-PL are presented in Fig. 1A, B. High correlations were found between the TIS-PL and FMA-UE-PL (rho = 0.71) total scores and the TIS-PL and FMA-LE-PL (rho = 0.76) total scores (see Tables I and II). Similarly, the correlations between the Dynamic sitting balance subscale score and the FMA-UE-PL total score, as well as between the Coordination subscale score and the FMA-LE-PL total score, were high. Moderate correlations were found between the Coordination subscale score and the FMA-UE-PL total score, as well as between the Dynamic sitting balance subscale score and the FMA-LE-PL total score. The correlations between the Static sitting balance subscale score and the FMA-UE-PL and FMA-LE-PL total scores were low. Based on the absolute scoring method, 6 out of 8 hypotheses for the TIS-PL were confirmed (75%), indicating moderate construct validity (Tables I and II).

The relationships between the TIS-PL and ARAT-PL scores are presented in Fig. 1C. The correlation between the TIS-PL and the ARAT-PL for absolute scores was moderate (rho = 0.61). A low-to-high correlation was observed between the TIS-PL and ARAT-PL subscale scores (Table X).

**Table X:** Analysis of correlation to assess the association between the TIS-PL and ARAT-PL scores (*n* = 80)

ARAT-PL	TIS-PL	R	P
Grasp	Static sitting balance	0.46	0.001
Grasp	Dynamic sitting balance	0.75	< 0.001
Grasp	Co-ordination	0.57	< 0.001
Grasp	Total TIS-PL	0.68	< 0.001
Grip	Static sitting balance	0.40	0.026
Grip	Dynamic sitting balance	0.73	< 0.001
Grip	Co-ordination	0.52	< 0.001
Grip	Total TIS-PL	0.63	< 0.001
Pinch	Static sitting balance	0.38	0.020
Pinch	Dynamic sitting balance	0.66	< 0.001
Pinch	Co-ordination	0.49	< 0.001
Pinch	Total TIS-PL	0.59	< 0.001
Gross Movement	Static sitting balance	0.47	0.001
Gross Movement	Dynamic sitting balance	0.72	< 0.001
Gross Movement	Co-ordination	0.54	< 0.001
Gross Movement	Total TIS-PL	0.65	< 0.001
Total ARAT-PL	Static sitting balance	0.39	0.012
Total ARAT-PL	Dynamic sitting balance	0.68	< 0.001
Total ARAT-PL	Co-ordination	0.50	< 0.001
Total ARAT-PL	Total TIS-PL	0.61	< 0.001

ARAT-PL: Polish version of the Action Research Arm Test; TIS-PL: Polish version of the Trunk Impairment Scale; R: Spearman rank correlation coefficient; P: probability value.

### Floor and ceiling effects

The TIS-PL showed a significant ceiling effect, affecting 46% of the participants evaluated. No floor effect was detected (0%).

## DISCUSSION

The purpose of this study was to determine the reliability and validity of the culturally adapted Polish version of the TIS. The results indicated that the TIS-PL demonstrated moderate to excellent reliability and moderate construct validity. Additionally, a moderate relationship was generally observed between trunk function, as assessed by the TIS-PL, and upper extremity activity, as measured by the ARAT-PL, in individuals after stroke. However, high positive correlations were observed between dynamic trunk postural control and fine motor skills of the hand and fingers in stroke patients.

### Reliability

In this study, excellent test–retest reliability was found for the total and subscale scores TIS-PL. Similar results have been reported in the literature by Verheyden et al. (12) and Sağ (17), who presented ICC coefficients ranging from 0.96 to 1 for the test–retest reliability of this instrument. Other studies (13, 15, 16) have demonstrated good to high test–retest reliability indices, ranging from 0.67 to 0.98, as assessed by Cohen’s kappa coefficients. The inter-rater reliability of the TIS-PL as per Cohen’s kappa coefficient and the ICC was perfect. The degree of agreement among independent raters was moderate for 2 items and good for 1 item. The inter-rater reliability scores from previous studies vary widely, from 0.49 to 0.99 (қ, ICC) (13, 15, 16). However, excellent inter-rater reliability has been reported for the original version of the scale (12).

The Cohen’s kappa coefficient indicated an almost perfect level of agreement, and the interobserver agreement, measured via percentage agreement, was ≥0.90. Thus, the results provide evidence that the Polish version of the TIS demonstrates moderate to excellent reliability, comparable to that of the original version. Moreover, our findings appear robust, as all previous studies included substantially smaller sample sizes than ours.

### Internal consistency

The total and subscale scores of the TIS-PL presented excellent internal consistency. The Cronbach’s alpha values for the Static sitting balance, Dynamic sitting balance, Coordination, and total TIS-PL scores were 0.90, 0.85, 0.91, and 0.89, respectively. These results are consistent with those reported in previous studies, which ranged from α = 0.61 to 0.97 (12, 13, 15, 16). Our findings indicate that the Polish version of TIS has been accurately translated and exhibits consistency comparable to the original scale. Notably, for the Coordination subscale, the TIS-PL results showed slightly higher internal consistency than the original version reported by Verheyden et al. (12).

### Validity

The construct validity of the TIS-PL was established. Correlation coefficients were calculated for TIS-PL, FMA-UE-PL, and FMA-LE-PL. High correlation was observed between the total scores of the scales, with coefficients of 0.71 and 0.76, respectively. However, different patterns emerged across the subscales. Correlations between the TIS-PL subtests and FMA-UE-PL ranged from low to high (R = 0.42 to 0.72). A slightly lower correlation was found between the TIS-PL subtests and FMA-LE-PL, indicating low to moderate relationships (R = 0.39–0.69). Our findings suggest that the strongest correlation was observed with lower extremity function, while correlations with upper extremity function were slightly weaker. After analysing all the predefined hypotheses, the TIS-PL demonstrated moderate construct validity. Our choice of comparator tools was guided by the functional relationship between trunk and limb movement (4, 19, 20, 39, 40, 42–47) and the availability of a validated Polish version of the outcome measure. To our knowledge, the construct validity of the TIS has been tested using hypothesis-driven methods in only 1 other study (48). That study reported acceptable correlations (R > 0.4) using Pearson’s coefficients between the TIS and the Barthel Index, the Trunk Control Test, and the Functional Independence Measure. Another study (1) reported good construct validity; however, a different method – Confirmatory Factor Analyses – was employed.

### Relationship between trunk function and upper extremity activity

The strength of the correlation between the TIS-PL and ARAT-PL scores was calculated. According to the literature, there is a relationship between the level of trunk postural control and upper extremity functional activity in patients after stroke. In this study, high to moderate positive correlations were found between the total and partial scores of the TIS-PL and ARAT-PL assessments. In a few cases, low positive correlations were observed between specific subscale scores of the 2 outcome measures. A high correlation was found between the Dynamic sitting balance subscale of the TIS-PL and the Grasp, Grip, and Global movement subtests of the ARAT-PL (rho = 0.75, 0.73, and 0.72, respectively). Moderate correlations (rho = 0.50–0.57) were observed between the Coordination subscale of the TIS-PL and all ARAT-PL subtests. The lowest correlations (rho = 0.38–0.50) were observed for the Static sitting balance subscale, indicating weak relationships.

To our knowledge, no previous study has reported a correlation between upper-extremity activity assessed with the ARAT and trunk postural control assessed with the TIS. In addition, the literature does not present such detailed analyses of correlation relationships between the results of individual subtests of both outcome measures. The relationship between the level of impairment in the upper extremity and trunk in stroke was presented by Likhi et al. (4). Trunk control was evaluated using the TIS, and upper extremity functional performance was evaluated using the Simplified Stroke Rehabilitation Assessment of Movement. A lower correlation was observed than that in this study (rho = 0.50-0.60). Another study compared the results of the TIS and the FMA-UE. A moderate relationship (rho = 0.53, rho = 0.67) was found between the results obtained in these assessments (37, 38). In this case, the tested compound was at a lower level than ours. Thus, the results presented in this work indicate a stronger relationship between individual trunk control skills and various upper extremity functional skills in stroke than has been shown in previous studies. In particular, the results of the TIS-PL dynamic sitting balance subtest are highly correlated with the ability to perform various types of grips. This demonstrates a strong connection between upper-limb gross and fine motor control and trunk posture control in the sitting position in people with stroke. Moreover, a high correlation was observed between contralateral trunk movements and upper-limb function. This may indicate a relationship between the degree of trunk muscle tension on one side and the functional efficiency of the upper limb (gripping and manipulating the hand) on the opposite side. Although one of the conditions for standardizing measurements during the ARAT test was to maintain the trunk resting on the chair, the present study found that trunk control is essential when using the upper extremities, even in a propped position.

Trunk control provides the fundamental proximal stability required for efficient and coordinated upper limb movements. Furthermore, stronger trunk function is associated with better upper limb performance and recovery in both healthy individuals and stroke survivors (41–45).

### Floor and ceiling effect

No floor effect was observed in our study; however, a significant ceiling effect was present, affecting 46% of participants. These findings suggest that a substantial proportion of the participants experienced little difficulty in completing the tasks. The Coordination subscale was the only component that posed greater demands on the participants. It appears that TIS-PL is an outcome measure that may not effectively differentiate patients with minimal trunk impairment and is more suitable for assessing individuals with moderate to severe trunk dysfunction. This observation may indicate that, for some subscales, the TIS scoring system is not sufficiently sensitive to detect subtle deficits in patients who have largely recovered after a stroke or who exhibit mild trunk impairment.

### Strengths of the study

The present study provides the first validated Polish version of a standardized tool for assessing trunk control after stroke, addressing a gap in Polish neurorehabilitation research and practice. The translation and cross-cultural adaptation followed international guidelines, ensuring conceptual and linguistic accuracy. Psychometric testing demonstrated excellent reliability and moderate validity, confirming that the scale is both consistent and clinically relevant. Moreover, the instrument is brief and practical, making it feasible for routine clinical and research use.

### Limitations

This study has several limitations. First, although the sample size was adequate for preliminary validation, it was relatively small and limited to a single clinical population, potentially limiting generalizability. Second, the study did not include a longitudinal assessment to evaluate responsiveness or sensitivity to change over time. Third, the sample was heterogeneous in terms of the type of rehabilitation participants were undergoing. Moreover, many patients demonstrated a relatively high level of functional control of the trunk and upper limbs, resulting in a ceiling effect. This likely restricted score variability, reduced measurement error, and inflated both intra- and inter-rater reliability estimates. Consequently, this exaggerated reliability may also have affected our validity measures, as the TIS failed to discriminate between many individuals. Future research should therefore include individuals with moderate and low levels of motor impairment following stroke to better evaluate the discriminatory power of the TIS-PL. Additionally, the relevance of this research is currently restricted to the Polish population, and comparable studies are recommended for other nations. Finally, the lack of a culturally adapted Polish version of a trunk control scale represents a methodological limitation; however, the use of the ARAT and FMA was reasonable given the instrument’s unavailability.

### Conclusion

The Polish version of the TIS-PL demonstrates almost perfect reliability and moderate construct validity. Our results support the clinical and research use of the TIS-PL as a valid outcome measure for assessing trunk impairment in the Polish post-stroke population. Furthermore, the novel finding is that a higher level of dynamic trunk postural control is associated with greater fine motor skills of the hand and fingers in stroke patients.
